# Association Between Serum Uric Acid Levels and Traditional Cardiovascular Risk Factors in Xiamen Residents of China: A Real-World Study

**DOI:** 10.3389/fcvm.2022.913437

**Published:** 2022-05-17

**Authors:** Peng Zhang, Linjian Chen, Zhaokai Li, Wei Ni, Lin Wang, Wanchun Mei, Guoqiang Ruan, Zaixing Shi, Cuilian Dai

**Affiliations:** ^1^Department of Cardiology, Xiamen Cardiovascular Hospital of Xiamen University, School of Medicine, Xiamen University, Xiamen, China; ^2^Xiamen Key Laboratory of Cardiovascular Disease, Xiamen Cardiovascular Hospital of Xiamen University, School of Medicine, Xiamen University, Xiamen, China; ^3^Key Laboratory of Health Technology Assessment of Fujian Province, School of Public Health, Xiamen University, Xiamen, China

**Keywords:** serum uric acid, cardiovascular disease, risk factor, china-pAR risk score, xiamen

## Abstract

**Background:**

Serum uric acid (SUA) levels was associated with cardiovascular diseases and cardiovascular events. However, the relationship between SUA levels and traditional cardiovascular risk factors has not been well-established among Xiamen residents. Our study aimed to estimate the relationship between SUA levels and cardiovascular risk factors among Xiamen residents using real-world data.

**Methods:**

Participants were enrolled from eight community health service centers in Xiamen, China. Participants were divided into four groups according to quartiles of the SUA levels. The history of diseases, the use of medications and the levels of laboratory parameters were collected. The China-PAR equation was used to evaluate the 10-year atherosclerotic cardiovascular disease (ASCVD) risk.

**Results:**

A total of 1,322 participants were enrolled. About 568 (43.0%) were men and 754 (57.0%) were women. The prevalences of hypertension, elderly, current smokers, and obesity were higher in the quartile 4 (Q4) group than the quartile 1 (Q1) group (all *p* < 0.001). Multivariable logistic regression analysis showed the OR for hypertension was 2.671 (95% CI 1.777–4.015, *p* < 0.001) in the Q4 group compared with that in the Q1 group. Further logistic regression showed the OR for hypertension was 3.254 (95% CI 1.756–6.031, *p* < 0.001) in men and 2.314 (95% CI 1.354–3.955, *p* = 0.002) in women in the Q4 group compared with that in the Q1 group, respectively. In addition, the percentage of participants with low 10-year ASCVD risk calculated by China-PAR was higher in the Q1 group than that in the Q4 group (55.86 vs. 31.82%, *p* < 0.001). The percentage of participants with high 10-year ASCVD risk was lower in the Q1 group compared with the Q4 group (15.32 vs. 25.45%, *p* < 0.001). Multiple linear logistic regression showed the 10-year China-PAR ASCVD risk scores was positively correlated with SUA after adjusting for various factors (β = 0.135, *p* = 0.001).

**Conclusion:**

Serum uric acid was associated with several cardiovascular risk factors in Xiamen residents. The percentage of high 10-year ASDVD risk was higher in participants with hyperuricemia. Participants with hyperuricemia may experience cardiovascular benefit from uric acid-lowering therapy.

## Introduction

Uric acid (UA) is the final product of purine degradation, has essential antioxidant effects in human body (1, 2). UA could not be further catabolized into allantoin because of the mutations of the gene encoding uricase during evolution. When serum uric acid (SUA) levels exceed urate solubility, i.e., at approximately 6.8 mg/dL, monosodium urate crystal will form and be deposited in tissues. Increased SUA was reported to serve as a pro-oxidant agent and promote oxidative stress (3). Hyperuricemia is not only a cause of gout but a contributor to the development cardiovascular diseases (CVD), diabetes and chronic kidney disease (4).

Epidemiological studies have shown the positive correlation between hyperuricemia and cardiovascular diseases, including hypertension, atherosclerosis, atrial fibrillation, and heart failure (5). It has been reported hyperuricemia was associated with increased risk of cardiovascular events. Evidence also suggested elevated SUA has relations with traditional cardiovascular risk factors such as hypertension, insulin resistance, obesity, and hyperlipidemia (6). However, the association between SUA and CVD and established cardiovascular risk factors are controversial.

The 10-year atherosclerotic cardiovascular disease (ASCVD) risk prediction model derived from the China-PAR project was validated in Chinese population (7, 8). After calculating the China-PAR 10-year ASCVD risk scores, the participants will be classified into low-, moderate-, and high-risk for ASCVD. Based on the interaction between SUA and cardiovascular disease and cardiovascular events, we suspected that SUA may associated with the predicted 10-year ASCVD risk. However, data on the relationship between SUA and the predicted 10-year ASCVD risk is limited.

Therefore, the aim of this study is to estimate the relationship between SUA levels and traditional cardiovascular risk factors and 10-year ASCVD risk based on real-world data among Xiamen residents.

## Materials and Methods

### Study Design and Participants

Participants were enrolled from eight community health service centers in Xiamen, China from November 2018 to June 2019. The inclusion criteria were an age of ≥18 years, continued residence in Xiamen for more than 2 years, and the provision of signed informed consent. Subjects with established ASCVD, inability or unwillingness to comply with the study requirements were excluded from this study. No uric acid-lowering agents were used in the enrolled subjects. Hyperuricemia was defined as baseline SUA levels >7 mg/dL (420 μmol/L) in men or >6 mg/dL (360 μmol/L) in women (9). Participants were divided into four groups according to quartiles of the SUA levels: quartile 1 (Q1) group (SUA ≤ 4.78 mg/dL, *n* = 333), quartile 2 (Q2) group (SUA 4.78–5.83 mg/dL, *n* = 329), quartile 3 (Q3) group (SUA 5.83–6.86 mg/dL, *n* = 330), and quartile 4 (Q4) group (SUA ≥ 6.86 mg/dL, *n* = 330). The protocol was approved by the Ethics Committee of Xiamen Cardiovascular Hospital of Xiamen University (approved number: 2018YLK3) and registered at Chinese Clinical Trial Registry (registration number: ChiCTR2200055327). Before recruitment, written informed consent was obtained from each participant.

### Assessment of Cardiovascular Risk Factors

For each participant, cardiovascular risk factors such as history of hypertension, diabetes mellitus, obesity and smoking were collected. The body weight index (BMI), blood pressure, blood lipid and blood glucose were measured. Hypertension was defined as systolic blood pressure ≥140 mmHg and/or diastolic blood pressure ≥90 mmHg or with anti-hypertensive treatment. Diabetes mellitus was defined as fasting plasma glucose (FPG) ≥ 7.0mmol/L or HbA1c ≥ 6.5%. Obesity was defined as BMI ≥ 28.0 kg/m^2^ (10). Abdominal obesity was defined as waist circumference (WC) ≥ 90 cm in men and ≥85 cm in women (11).

### Cardiovascular Risk Estimation Based on China-PAR Project

The China-PAR 10-year ASCVD risk score has been developed to estimate the risk of ASCVD (7). The China-PAR equation included the following variables: sex, age, treated or untreated systolic BP (SBP), total cholesterol (TC), high-density lipoprotein cholesterol (HDL-C), WC, current smoking status, diabetes mellitus, geographic region, urbanization, and family history of ASCVD. Based on the scores, the subjects were split into three risk categories: <5% as low-risk, 5–10% as moderate-risk, and ≥10% as high-risk.

### Statistical Analyses

Normally distributed continuous variables are reported as the mean ± SD and were compared using the paired or unpaired Student’s *t*-test. Non-normally distributed continuous variables are expressed as the medians with interquartile ranges and were determined by the Wilcoxon signed-rank test or Mann–Whitney U test. Categorical variables are presented as the counts and percentages and were evaluated by the χ^2^-test or Fisher’s exact test as appropriate. *P* < 0.05 was considered to be statistically significant. Statistical analyses were conducted with SPSS version 20.0 software (IBM, Armonk, NY, United States).

## Results

### Characteristics of the Participants

A total of 1,322 participants were enrolled. For all the participants, the average age was 66 years old, with male gender distribution of 43.0%. Among SUA quartiles groups, age, WC, serum creatinine, TG, and the percentage of current smoking and hypertension were significantly different between the Q1 group and the Q4 group (all *p* < 0.001). The clinical characteristics of the study participants are shown in Table 1.

**TABLE 1:** Clinical characteristics of participants according to quartiles of serum uric acid.

Variables	All the participants (*n* = 1,322)	Q1 group (SUA ≤ 4.78 mg/dL, *n* = 333)	Q2 group (SUA 4.78–5.83 mg/dL, n = 329)	Q3 group (SUA 5.83–6.86 mg/dL, n = 330)	Q4 group (SUA ≥ 6.86 mg/dL, *n* = 330)	*P* value
Age, years	66.00 (54.75–71.00)	63.00 (48.50–70.00)	66.00 (54.50–71.00)	68.00 (58.00–73.00)	67.00 (57.00–73.00)	<0.001*
WC, cm	84.00 (78.00–90.00)	81.00 (75.00–87.00)	83.00 (78.00–88.00)	85.00 (80.00–90.00)	86.50 (81.00–93.00)	< 0.001*
Male,%	568 (43.0)	75 (22.5)	115 (35.0)	158 (47.9)	220 (66.7)	<0.001*
Smoke,%	192 (14.5)	34 (10.2)	37 (11.2)	48 (14.5)	73 (22.1)	<0.001*
Heart rate, bpm	72.00 (66.00–78.00)	72.00 (66.00–79.00)	72.00 (66.00–78.00)	72.00 (66.00–78.00)	72.00 (66.75–80.00)	0.780
Hypertension,%	627 (47.4)	110 (33.0)	143 (22.8)	169 (51.2)	205 (62.1)	<0.001*
Dyslipidemia,%	442 (33.4)	99 (29.7)	113 (34.3)	118 (35.8)	112 (33.9)	0.385
Hyperuricemia,%	431 (32.6)	0 (0)	0 (0)	128 (29.7)	303 (70.3)	<0.001*
Diabetes,%	436 (33.0)	101 (30.3)	113 (34.3)	118 (35.8)	104 (31.5)	0.280
Hemoglobin, g/L	137.00 (128.00–148.00)	132.00 (126.00–140.00)	135.00 (128.00–146.00)	138.00 (130.00–150.00)	141.00 (131.75–152.00)	<0.001*
SUA, mg/dL	5.83 (4.79–6.86)	4.26 (3.60–4.50)	5.11 (5.35–5.58)	6.36 (6.07–6.59)	7.70 (7.21–8.57)	<0.001*
Serum creatinine, μmol/L	70.50 (59.68–84.00)	64.00 (54.95–75.86)	67.10 (58.00–77.00)	71.93 (61.16–84.70)	80.00 (68.10–91.84)	<0.001*
eGFR^†^, mL/min/1.73 m^2^	78.70 (62.50–97.00)	79.20 (62.00–97.00)	80.90 (64.80–100.00)	78.00 (64.05–94.75)	76.90 (59.60–97.00)	0.212
TG, mmol/L	1.30 (0.90–1.83)	1.03 (0.80–1.50)	1.30 (0.88–1.81)	1.37 (1.00–1.91)	1.49 (1.01–2.09)	<0.001*
TC, mmol/L	5.05 (4.38–5.80)	5.04 (4.31–5.85)	5.07 (4.27–5.72)	5.05 (4.45–5.91)	5.08 (4.45–5.79)	0.696
LDL-C, mmol/L	2.89 (2.32–3.52)	2.90 (2.28–3.47)	2.84 (2.23–3.48)	2.86 (2.35–3.52)	2.94 (2.43–3.58)	0.185
Glucose, mmol/L	5.76 (5.13–7.13)	5.59 (5.04–6.97)	5.81 (5.14–7.37)	5.70 (5.12–7.20)	5.90 (5.28–6.98)	0.124
HbA1c,%	6.70 (5.90–9.10)	6.40 (5.63–8.18)	7.15 (5.96–10.72)	6.75 (6.20–10.48)	6.70 (5.80–8.85)	0.051

Normally distributed continuous variables are presented as the mean ± SD and non-normally distributed continuous variables are expressed as the medians with interquartile ranges. Categorical variables are reported as the counts and percentages. SUA, serum uric acid; WC, waist circumference; eGFR, estimated glomerular filtration rate; TG, triglyceride; TC, total cholesterol; LDL-C, low-density lipoprotein cholesterol. *p < 0.05 (the Q4 group vs. the Q1 group).

### Association Between Serum Uric Acid Levels and Cardiovascular Risk Factors

**FIGURE 1:**
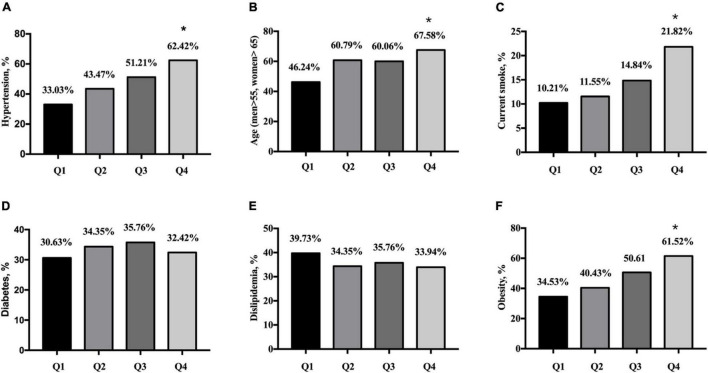
Prevalence of cardiovascular disease risk factors according to quartiles of serum uric acid. **(A)** Prevalence of hypertension in quartiles of SUA. **(B)** Prevalence of age in quartiles of SUA. **(C)** Percentage of smoker in quartiles of SUA. **(D)** Prevalence of diabetes mellitus in quartiles of SUA. **(E)** Prevalence of dislipidemia in quartiles of SUA. **(F)** Prevalence of obesity in quartiles of SUA. Q1: SUA ≤ 4.78 mg/dL, *n* = 333; Q2: SUA 4.78–5.83 mg/dL, *n* = 329; Q3: SUA 5.83–6.86 mg/dL, *n* = 330; Q4: SUA ≥ 6.86 mg/dL, *n* = 330. SUA, serum uric acid. *p < 0.05 (the Q4 group vs. the Q1 group).

Among the participants, 628 had hypertension (47.5%). The prevalences of hypertension, elderly, current smokers and obesity were higher in the Q4 group than the Q1 group (all *p* < 0.001, Figure 1). The percentages of diabetes and dyslipidemia were not significantly different between the Q4 group than that in the Q1 group (both *p* > 0.05, Figure 1).

Multivariable logistic regression analysis was performed to assess the relation between SUA and cardiovascular risk factors. As shown in Table 2, the OR for WC, male and hypertension was 1.044 (95% CI 1.022–1.067, *p* < 0.001), 0.146 (95% CI 0.089–0.238, *p* < 0.001), 2.671 (95% CI 1.777–4.015, *p* < 0.001) in the Q4 group compared with that in the Q1 group respectively, which showed that hyperuricemia was positively correlated with WC and hypertension, but negatively correlated with male.

**TABLE 2:** Association between serum uric acid and cardiovascular risk factors.

Variable	OR (95%CI)	*P* value
Age	1.012 (0.997–1.028)	0.109
WC	1.044 (1.022–1.067)	<0.001
Male	0.146 (0.089–0.238)	<0.001
Smoke	0.581 (0.331–1.018)	0.058
Hypertension	2.671 (1.777–4.015)	<0.001
Hemoglobin	1.002 (0.991–1.013)	0.737
Serum creatinine	1.003 (0.995–1.012)	0.457
TG	1.103 (0.975–1.246)	0.118

*WC, waist circumference; TG, triglyceride.*

### Relationship Between Serum Uric Acid Levels and Cardiovascular Risk Factors According to Gender Stratification

Among the participants, 568 (43.0%) were men and 754 (57.0%). The prevalence of hyperuricemia was 34.0% in men and 31.6% in women (*p* = 0.354). Multivariable logistic regression was further analyzed according to gender stratification. As shown in Table 3, the OR for hypertension was 3.254 (95% CI 1.756–6.031, *p* < 0.001) in the Q4 group compared with that in the Q1 group in men. The OR for age, waist, and hypertension was 1.045 (95% CI 1.020–1.071, *p* < 0.001), 1.048 (95% CI 1.017–1.079, *p* = 0.002), 2.314 (95% CI 1.354–3.955, *p* = 0.002) in the Q4 group compared with that in the Q1 group respectively in women. The multivariable logistic regression analysis according to gender stratification showed hyperuricemia positively correlated with hypertension in both genders and positively correlated with age and WC in women.

**TABLE 3:** Association between serum uric acid and cardiovascular risk factors according to gender stratification.

	Men		Women	
		
Variables	OR (95%CI)	*P* value	OR (95%CI)	*P* value
Age	0.981 (0.959–1.003)	0.093	1.045 (1.020–1.071)	<0.001
WC	1.031 (0.996–1.067)	0.088	1.048 (1.017–1.079)	0.002
Smoke	0.629 (0.350–1.132)	0.122	1.123 (0.196–7.504)	0.836
Hypertension	3.254 (1.756–6.031)	<0.001	2.314 (1.354–3.955)	0.002
Hemoglobin	1.001 (0.980–1.022)	0.928	1.001 (0.988–1.015)	0.846
Scr	1.000 (0.993–1.008)	0.892	1.009 (0.994–1.025)	0.243
TG	1.057 (0.827–1.350)	0.658	1.124 (0.989–1.278)	0.073

*WC, waist circumference; TG, triglyceride.*

### Association of SUA With 10-Year ASCVD Risk

**FIGURE 2:**
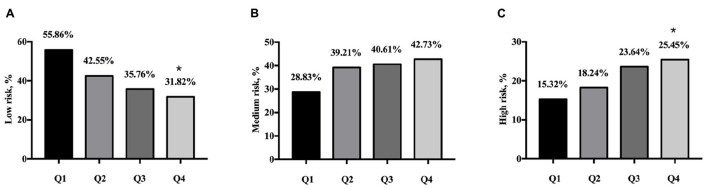
Ten-year ASCVD risk categories by China-PAR according to quartiles of serum uric acid. **(A)** Percentage of low risk in quartiles of SUA. **(B)** Percentage of medium risk in quartiles of SUA. **(C)** Percentage of high risk in quartiles of SUA. Q1: SUA ≤ 4.78 mg/dL, *n* = 333; Q2: SUA 4.78–5.83 mg/dL, *n* = 329; Q3: SUA 5.83–6.86 mg/dL, *n* = 330; Q4: SUA ≥ 6.86 mg/dL, *n* = 330. ASCVD, atherosclerotic cardiovascular disease; SUA, serum uric acid. *p < 0.05 (the Q4 group vs. the Q1 group).

The 10-year ASCVD risk was calculated by China-PAR in this study. There were 548 (41.5%) participants divided into low-risk group, 500 (37.8%) divided into medium-risk group, 273 (20.7%) divided into high-risk group. As shown in Figure 2, the percentage of participants with low risk of 10-year ASCVD were higher in the Q1 group than that in the Q4 group (55.86 vs. 31.82%, *p* < 0.001). The percentage of participants with high risk of 10-year ASCVD were lower in the Q1 group compared with the Q4 group (15.32 vs. 25.45%, *p* < 0.001, Figure 2).

Multiple linear logistic regression was then analyzed. As shown in Table 4, **10**-year China-PAR ASCVD risk scores was positively correlated with SUA after adjusting for various factors (β = 0.135, *p* = 0.001).

**TABLE 4:** Association of 10-year China-PAR ASCVD risk scores with serum uric acid.

Variables	Beta coefficients	*P* value
WC	0.249	<0.001
Serum creatinine	0.129	0.001
TG	0.082	0.026
10-Year China-PAR ASCVD Risk Scores	0.135	0.001

*ASCVD, atherosclerotic cardiovascular disease; WC, waist circumference; TG, triglyceride.*

## Discussion

The mortality of ASCVD is increasing gradually in China in part due to the increased exposure to multiple cardiovascular risk factors (12). Control of risk factors in the secondary prevention of ASCVD could reduce the risk of cardiovascular events (13). Elevated SUA was reported to play roles in the development in ASCVD and multiple cardiovascular risk factors (6, 14). In addition, several clinical investigations showed increased SUA might be a predictive marker for cardiovascular outcomes (15). Although the causality between hyperuricemia and ASCVD remains controversial, a growing interest has been paid in that relationship because of the increased prevalence of hyperuricemia in the world. Our real-world cross-sectional study found that increased SUA levels was significantly associated with several traditional cardiovascular risk factors and the 10-year ASCVD risk among Xiamen residents.

The estimated prevalence of hyperuricemia among Chinese adults was 8.4% in 2009–2010 and 14.0% in 2018–2019 (16, 17). In Shanghai population, the prevalence of hyperuricemia was 17.2% (22.2% in men and 10.8% in women) in 2015 (18). In Jiangsu adults, the prevalence of hyperuricemia was 13.3% (16.9% in men and 10.3% in women) in 2015 (19). According to our real-world study, the prevalence of hyperuricemia was 32.6% (34.0% in men and 31.6% in women) among Xiamen population (Table 1), which was higher than that in eastern Chinese population in 2015 and in Chinese adults in 2018–2019 (17–19). Changes in lifestyle, eating habits and environmental factors are attributed to the development of hyperuricemia (20). In addition, the genetic mutations or polymorphisms related to the process of UA metabolism may partly explain this phenomenon (21, 22).

Numerous studies have showed the association between SUA and hypertension (5). Modest hypertension was found in the hyperuricemia rat model induced by potassium oxonate, which may be due to oxidative stress, decreased nitric oxide availability and activated renin–angiotensin system (23). Hyperuricemia was common in patients with hypertension, especially in those with accelerated or malignant hypertension (24). In addition, several studies have linked hyperuricemia to non-dipping pattern of hypertension (25, 26). Evidence suggested that elevated SUA increased the risk of incident hypertension independently (27, 28). As the evidence accumulated, SUA was listed as a factor influencing cardiovascular risk in hypertensive patients according to the 2018 European Society of Cardiology (ESC) and European Society of Hypertension (ESH) guidelines for the management of arterial hypertension (29). In our study, SUA was associated with hypertension in both the entire individuals (2.671, 95% CI 1.777–4.015) and gender subgroups (3.254, 95% CI 1.756–6.031 for men; 2.314, 95% CI 1.354–3.955 for women).

The 10-year ASCVD risk score based on China-PAR project have been proved to be validated in predicting ASCVD among Chinese population (7). Participants will be divided into three risk categories based on the China-PAR equations (<5%: low risk; 5–10%: medium risk; ≥10%: high risk). In our study, the 10-year ASCVD risk of each participant was assessed by the China-PAR equations. It is worth noting that the medium and high risk of 10-year ASCVD was 58.5% of participants. A higher proportion of high 10-year ASCVD risk was found in participants with hyperuricemia. SUA was positively related with China-PAR scores in Xiamen residents. Our data suggested SUA was significantly associated with 10-year ASCVD risk. The predictive role of SUA in 10-year ASCVD risk needs to be explored in further investigation.

Although this study highlights the association of SUA with cardiovascular risk factors and 10-year ASCVD risk in Xiamen residents, there are several limitations. The participants in this study were drawn from only 1 region in China, which may not reflect the national situations. Data on broad lifestyle changes such as exercise, stress relief and diet were not collected. Further large-scale study evaluating the role of SUA in cardiovascular diseases are warranted.

In summary, SUA was associated with several cardiovascular risk factors in Xiamen residents. The risk of high 10-year ASDVD was higher in participants with hyperuricemia. Uric acid-lowering therapy is needed to optimize risk factor control of ASCVD in Chinese adults with hyperuricemia.

## Data Availability Statement

The raw data supporting the conclusions of this article will be made available by the authors, without undue reservation.

## Ethics Statement

The studies involving human participants were reviewed and approved by Ethics Committee of Xiamen Cardiovascular Hospital of Xiamen University. The patients/participants provided their written informed consent to participate in this study.

## Author Contributions

CD have conceived and designed the study. LC, ZL, WN, LW, and WM participated in the data collection. GR, ZS, and CD provided the supervision support. PZ and LC participated in drafting the manuscript. PZ and ZS performed data analysis. All authors contributed to the critical revisions and final approval of the manuscript.

## Conflict of Interest

The authors declare that the research was conducted in the absence of any commercial or financial relationships that could be construed as a potential conflict of interest.

## Publisher’s Note

All claims expressed in this article are solely those of the authors and do not necessarily represent those of their affiliated organizations, or those of the publisher, the editors and the reviewers. Any product that may be evaluated in this article, or claim that may be made by its manufacturer, is not guaranteed or endorsed by the publisher.
